# Influence of nicotine on machined- and anodized-surface implants. Histometric analysis

**DOI:** 10.4317/jced.54127

**Published:** 2017-10-01

**Authors:** Maria-Salete-Sandini Linden, Luiz-Renato Paranhos, João-Paulo De Carli, Micheline-Sandini Trentin, Marcos-Eugênio de Bittencourt, Pâmela-Letícia Santos, Francisco-Carlos Groppo, Juliana-Camma Ramacciato

**Affiliations:** 1 DDS, MSc, PhD, Professor, Department of Dentistry. University of Passo Fundo, Passo Fundo, RS, Brazil; 2 Professor, Department of Dentistry. Federal University of Sergipe, Lagarto, SE, Brazil; 3 Professor, Oral Biology Postgraduate Program, Sagrado Coração University, Bauru, SP, Brazil; 4 Professor, Department of Physiological Sciences. State University of Campinas, Piracicaba, SP, Brazil; 5 Professor, Department of Dentistry. Faculty SL Mandic, Campinas, SP, Brazil

## Abstract

**Background:**

The nicotine may generate a influence on bone repair and longevity of dental implants. This fact makes studies to improve the surface of the implants are constantly conducted. This study aimed to evaluate the influence of subcutaneous nicotine injection in the osseointegration process on different implant surfaces, through histomorphometric analysis.

**Material and Methods:**

Therefore, twenty-two male rabbits were randomly distributed into two groups according to the subcutaneous injections: (1) nicotine, 3 mg/day/kg and (2) 0.9% NaCI, 3 mL/day/kg, three times a day. Subgroups were then designated - machined and anodized dental implants were installed in the right and left tibia bones, respectively. The animals were subjected to euthanasia after periods of eight weeks for histomorphometric analysis. The bone samples with implants were removed and the routine histological processing was performed. Next, the images obtained from the blades were evaluated by the Image Tool™ software, assessing the osseointegrated areas of implants (BIC), in pixels. Data obtained were subjected to intergroup statistical analysis through the Kruskal-Wallis non-parametric test (α=5%).

**Results:**

The test result showed no statistically significant difference among the groups studied (*p*=0.446).

**Conclusions:**

Based on the methodology studied, it is concluded that the daily application of low doses of nicotine did not interfere with the osseointegration of machined and anodized implants.

** Key words:**Bone-implant interface, implants, osseointegration.

## Introduction

Dental implants are predictable when bone quantity and quality are adequate. However, this is often not the case, such as in patients who smoke, where the areas of cancellous bone and lamellar cortical bone are thin (1).

Some studies report that nicotine may cause tissue ischemia and reduce vascular internal growth, and that these facts generate a negative influence on bone repair and longevity of dental implants (2-6).

Based on these challenges, implants are constantly being improved, especially regarding their surfaces, which favor higher adhesiveness of osteoblasts and consequent bone neoformation around implants, improving the prognosis for complex clinical situations (7-9). Anodized-surface implants have shown higher values of bone/implant contacts and of removal torque when compared to machined-surface implants (10-15).

Thus, the present study aimed to investigate, through the histometry in animal models, whether the use of daily nicotine injection for eight weeks may influence the osseointegration of titanium implants with treated (anodized) or untreated surface.

## Material and Methods

-Animals

The study was reviewed and approved by the Ethics Committee of the University of Passo Fundo (n.535/2006), RS, Brazil. Twenty-two (n=44 tibia) adult male New Zealand white rabbits (Oryctolagus cuniculus) weighing 3.5 to 4.0 kg, aged 8 to 10 months were used. All rabbits were housed in animal facilities at 25°C, in 12-hour light/dark cycles. Throughout the experimental period, the rabbits were housed in individual plastic cages and a normal chow diet and water were provided ad libitum.

The animals were randomly distributed into two groups: Control Group – animals received subcutaneous injection of 0.9% NaCI, 3 mL/Kg/day, three times a day for eight weeks (n=22); Test Group - animals received subcutaneous injections of 98% nicotine hydrogen tartrate salt (Sigma-Aldrich, Copenhagen, Denmark A/S), 3 mg/kg/day, three times a day (n=22) (15). Each group was then divided into two subgroups – machined and anodized dental implants – corresponding to the dental implant used, respectively.

-Surgical Procedure

The animals received atropine (0.50 mg – 0.44 mg/kg/i.p.), 15 minutes before surgery. General anesthesia was administered by an intramuscular injection of 2% xylazine hydrochloride, 5 mg/kg (Rompun; Bayer, São Paulo, Brazil) and 7mg/kg of tiletamine (zolazepan/zoletil – Virbac do Brasil Indústria e Comércio Ltda).

Experimental surgery for implant installation was performed as previously described (16). Then, trichotomy and antisepsis were performed in each tibia with iodine solution (10% PVPI, Riodeine Degermante, Rioquímica, SP, Brazil) and topical PVPI before surgical incision. Local anesthesia was performed by infiltrative injection with 2% mepivacaine (0.3 mL/kg, 2% Scandicaine™ with adrenalin 1:100.000, Septodont, France).

A careful surgical technique was performed with a 3-cm long incision on the proximal tibia just below the knee to the depth of the bone tissue. The soft tissue was carefully dissected and lifted with the aid of a periosteal elevator, exposing the bone tissue for implant insertion. After pre-threading the receptor site, implants were inserted in each proximal metaphysis, under 40 N of torque.

One machined-surface implant (Master Screw™) in the right tibia and one anodized-surface implant (Master Vulcano Actives™) in the left one were installed in each animal. Both implants had 3.75 mm of diameter, were 6-mm long, and screw-shaped with external hexagon (ASTM grade 4). Both implants were provided by Conexão Sistemas de Prótese (São Paulo, SP, Brazil). Soft tissues were replaced and sutured.

After suturing, intramuscular pentabiotic (0.1 mL/kg, Fort Dodge Saúde Animal Ltda, SP, Brazil) was performed immediately and at 5 days postoperatively. Sodium dipyrone (1 mg/kg/day, Ariston Indústrias Químicas e Farmacêuticas Ltda, São Paulo, SP, Brazil) was also administered. Neither food nor movement restriction was applied to the animals that remained in individual cag-es during the experimental period. All rabbits were euthanized by a lethal dose of pentobarbital (200 mg/kg) at 8 weeks after surgery.

-Histomorphometric analysis

The tissue samples (bone/implant interface) were removed and placed in 10% neutral buffered formalin. Then, these samples were dehydrated in increasing concentrations of ethanol (60-100%) and later infiltrated in light-curing resin (Technovit 7200 VLC, Kultzer Heraeus GmbH & Co., Wehrheim, Germany).

The blocks with implant and peri-implant bone tissue were cut in a central point through a cutting and wear system (Phenom Prox™, Anacom Científica, Araraquara, SP, Brazil). The blades were obtained with approximate thickness of 50 μm.

The images were analyzed in a light microscope (DIASTAR, Leica Reichert & Jung products, Germany) and captured through a Leica Microsystems DFC-300-FX digital camera (Leica Microsystems, Germany), with 1.3 megapixels of resolution, coupled to a regular light microscope and a computer.

**Figure 1:**
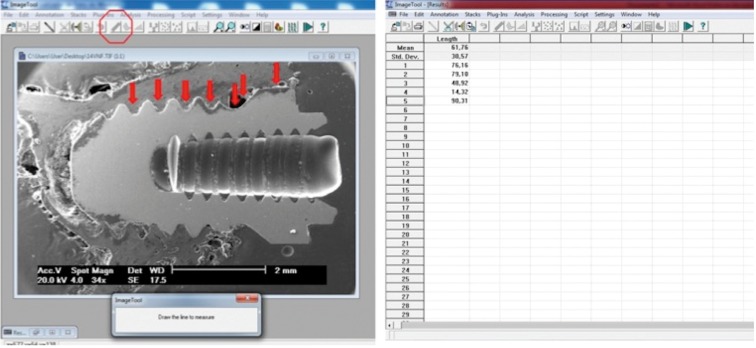
Image of a machined implant inserted in bone tissue that received nicotine, assessed in the Image Tool™ software (red arrows) by the “Distance” tool (circle), and bellow is the spreadsheet with automatically generated lengths, added up at the end of assessment.

The histometric analyses were performed with the image analysis software “Image Tool” (Fig. 1). The linear extension of bone tissue and implant surface (BIC) was calculated in pixels among the 3 most coronary loops (located in the cortical bone) in each side of the implant.

The BIC levels were compared intra- and intergroup. Data obtained in each type of comparison were subjected to statistical analysis through the Kruskal-Wallis non-parametric test (α=5%).

## Results

**Figure 2:**
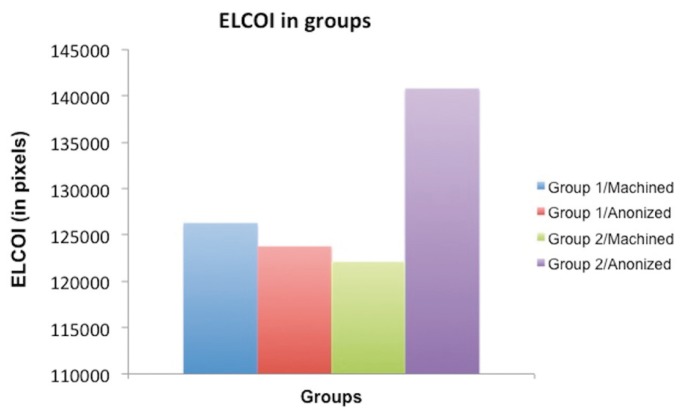
Graphic representation of mean values of BIC for each group (in pixels).

 Table 1 and Figure 2 show the perimeter of the osseointegrated implant surface for each group studied, in pixels, as well as mean and standard deviation. The Kruskal-Wallis non-parametric test was applied and showed no statistically significant difference among the groups studied (*p*=0.446). However, group 2/anodized was likely to higher osseointegration when compared to the other groups.

**Table 1:**
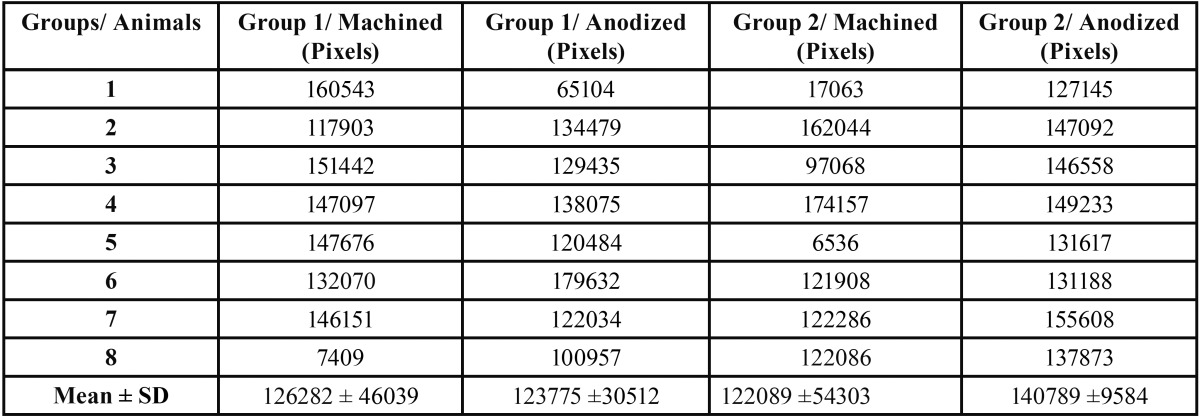
Perimeter of osseointegrated implant surfaces (in pixels) of the groups studied.

## Discussion

This study analyzed the influence of nicotine around osseointegrable implants, through histomorphometric analysis, on machined- and anodized-surface implants. The results showed that nicotine and implant surface had no negative effect on osseointegration.

These results corroborate experimental researches previously performed (1,17-19), which detected no negative impact of nicotine on the repair process. However, other authors (2-6) reported that nicotine causes tissue ischemia and reduces vascular internal growth with consequent negative effect on bone repair and the longevity of dental implants. Kallala *et al.* (20) confirm that the effect of nicotine is dose-dependent, meaning that it presents negative effects in high concentrations, while showing stimulant effects in low concentrations.

Although humans chronically inhale cigarette smoke and the bone tissue is exposed to its components for years, some authors (21-22) showed that studies in rabbits testing daily nicotine injections are relevant. Hence, César Neto *et al.* (23) compared only cigarette smoke and nicotine and found that the latter alone did not present the deleterious effects of the cigarette smoke, especially on medullary bone, concluding that the adverse effects of cigarette smoking on implant success may be reported only if the cigarette is considered along with all its components (24).

When considering other studies conducted in animals (1,17-18), it is found that they did not detect an impact on mineral bone density of female rats after two years of nicotine exposure. Such findings are added to the present study, which did not find differences in the osseointegration area among the groups that received and did not receive nicotine.

On the other hand, Kumar *et al.* (25) affirm that smoking is related to lower bone density and that the poor bone quality observed in smokers may lead to inadequate primary bone stability, resulting in excessive mobility and implant failure. However, their retrospective study showed no statistically significant difference of bone quality between smokers and non-smokers. Thus, we prevail by the findings by Akhter *et al.* (26), which hypothesized other tobacco agents as responsible for the decrease in bone density and increase in the risk of fracture observed in smokers.

Anodized implants are characterized by the presence of thick heterogeneous oxide, promoting a slightly increased bone response, especially in the first weeks after implantation, as affirmed by previous studies (10-11,14,27).

Peñarrocha *et al.* (28) showed that titanium implants with rough surface presented higher bone loss in smokers than in non-smokers, especially when installed in the maxilla. Corroborating this, Shibli *et al.* (29) performed a prospective study assessing the impact of smoking on bone-implant contact and bone density in treated and untreated implant areas, using anodized-surface implants, in humans. These authors concluded that the smoking habit in humans presents a deleterious effect on early bone response of the implants studied.

On the other hand, Kumar *et al.* (25) performed a research where the rates of bone loss were similar for implants with rough surface, both in smokers and non-smokers. As for Bain *et al.* (30), they found no differences between groups of smokers and non-smokers, monitoring machined-surface and textured-surface implants. Corroborating these studies, the present research found no influence of nicotine and implant surface on osseointegration.

From the aforementioned, it is agreed that basic scientific information on the bone repair mechanism, in response to implant installation, are always relevant. However, clinical studies are welcome to assess the long-term dynamic bone response using several materials and implant surfaces associated to risk factors such as smoking.

The subcutaneous application of low doses of nicotine had no negative influence on the osseointegration of machined and anodized implants.
